# Emergency medicine for 25 Years in Iceland – history of the specialty in a nutshell

**DOI:** 10.1186/s13049-017-0467-9

**Published:** 2018-01-03

**Authors:** Jón Baldursson, Hjalti Már Björnsson, Ari Palomäki

**Affiliations:** 1 Emergency Department, Landspítali G-2, Fossvogi, The National University Hospital of Iceland, IS-108 Reykjavík, Iceland; 2 Quality and Patient Safety Department, Landspítali – The National University Hospital of Iceland, Reykjavík, Iceland; 3 Department of Emergency Medicine, Mayo Clinic Health System, Austin/Albert Lea, MN USA; 4 Faculty of Medicine and Life Sciences, University of Tampere, Tampere, Finland; 5 Department of Emergency Medicine, Kanta-Häme Central Hospital, Hämeenlinna, Finland

## Abstract

After the early implementation of Emergency Medicine (EM) 25 years ago, Iceland became the first Nordic country to nationally realize the benefits of this specialty. However, the road has been rocky as in many other countries. The early years of EM in Iceland were characterized by a significant shortage of resources, particularly a lack of medical staff dedicated to EM and properly trained for the services required. The main task for the first couple of decades was to build the infrastructure of an operational emergency department based primarily on the model of EM. Although these efforts eventually led to a critical number of specialists becoming certified in EM, recruiting more people remains a priority in order to fully meet the need for specialty trained emergency physicians in Iceland. A key step towards achieving this goal was the initiation of a two-year residency program for specialty training in EM in year 2002. The program was based on a curriculum produced by the Icelandic Society for Emergency Medicine, which had been founded in year 2000. This training program is currently being redeveloped and the curriculum of the Royal College of Emergency Medicine in the UK will be adopted for use in Iceland. Another important milestone was the appointment of the first faculty member dedicated to EM at the University of Iceland. This created an opportunity to teach medical students EM and advance training at the graduate level. Also, conditions for scientific research in EM have been improved, following the establishment of an EM research institute in 2010.

Other Nordic countries may be able to benefit from lessons learned and experiences gained from the development of emergency medicine in Iceland during the past quarter of a century.

## Background

The Nordic countries have generally been slow to adopt the model of emergency medicine (EM) as a medical specialty to their health care systems. In this regard, their modernization of emergency care has been lagging behind the development in the United States (US), the United Kingdom (UK), Australia, New Zealand and other countries where EM has already been an intricate part of the health care systems for decades [1–3].

The first Nordic country to formally recognize EM as an independent specialty and start to incorporate the model of EM into the health care system was Iceland, where the first emergency physician (EP) was licensed as a specialist in 1992, a quarter of a century ago [4]. Recently, the specialty of EM has got its wings in other Nordic countries. EM became an independent specialty with a full-length training in Finland in 2013, in Sweden in 2015 [5–8]. The specialty will become legalized in Norway in 2018 [9]. Denmark on its side has four professors of EM, thus building academic foundations for the specialty, and the decision to adopt the specialty of EM was recently made by the Ministry of Health [10]. In a situation, where new countries are in the process of incorporating a new specialty into a Nordic health care system, it might be worthwhile to consider the lessons learned from this process over the last 25 years in Iceland [4].

The health care system in Iceland has a structure similar to some other Nordic countries, with the majority of services provided by hospitals and clinics owned and operated by the government. Private clinics also provide a significant proportion of patient care, but this is mostly funded by a single payer universal health insurance program run by the government.

Although the small nation of Iceland (about 333.000 inhabitants) has had a medical school since 1876 [11], until in recent years, limited opportunities have been available for post-graduate specialty training for physicians in Iceland. Essentially all Icelandic physicians have ventured abroad for their specialty training, with a majority seeking training in other Nordic countries but a significant number of physicians also receiving training in the US, the UK and several other countries. As most who receive their training abroad move back to Iceland to practice once their training is completed, the health care system in Iceland offers an interesting mix of different approaches. This has also created a culture in health care where new knowledge is often incorporated into the system by physicians who bring in new ideas and approaches which they have learned during their specialty training abroad. Consequently, from early on, the Ministry of Health had to create an efficient mechanism for the evaluation of credentials which Icelandic physicians had acquired from their training abroad. A systematic approach was necessary to ensure a proper basis for the certification of medical specialists in the Icelandic health care system. This official pathway of recognition eventually benefitted EM when the first, formally trained emergency physician applied for certification as a specialist.

## The early years

The first Icelandic physician, and to our knowledge the first from any Nordic country, to complete residency training in emergency medicine was Jón Baldursson, MD. After completing his training in the Department of Emergency Medicine at the University of Cincinnati Medical Center, Baldursson moved back to Iceland in 1991 to work in the Casualty Department at The Reykjavík City Hospital (Borgarspítalinn) which was then one of the three receiving hospitals for acute care in the capital region of the country. These three hospitals, i.e. Reykjavik City Hospital, Landspítali National Hospital and St. Joseph’s Hospital (Landakotsspítali) would finally merge in year 2000 to form the current Landspítali University Hospital. Already at this time the Casualty Department served as the main receiving unit for trauma and other emergencies. Casualty had been an independent department of the hospital since its inception in 1968, led mostly by orthopedic surgeons with a certain emphasis on minor trauma services. The unit was staffed with medical interns, occasionally with junior residents. Specialists were available on call for telephone consultation but usually not present on the shop floor. Other specialties, mainly internal medicine, also had a hand in on an on-call basis but did not provide on-site attending staff.

This lack of qualified and experienced emergency medical staff was bound to cause problems in Iceland as it had in other countries [1, 2]. In the late 1980s, both the public and the authorities openly expressed dissatisfaction with the poor quality of medical care provided in the Casualty Department, leading to the composition of “black reports” by two official committees. Both of these (the Ministry of Health of Iceland and the Board of Medical Directors in the Reykjavík City Hospital) concluded that this situation was unacceptable and had to be amended. This in turn led to the principal decision by the hospital’s leadership to institute specialist physician coverage in the Casualty Department 24 h every day of the year. Although only one of the newly hired staff was residency trained in EM, this decision, which became effective on 1 September 1991, was important in promoting the development of EM as a specialty in Iceland. It soon became evident that the only staff member who was at home in the Casualty Department was the EP who became certified as a specialist in EM by the Ministry of Health in 1992 [4]. For the orthopedic surgeons on the other hand, being tied up on the Casualty shop floor for two to three shifts a week would take them away from their orthopedic duties in the operating room, on the ward and in the clinic for a significant part of their time. It was no wonder that to them this arrangement proved to be a mere nuisance.

In the year 1994, a merger of Reykjavik City Hospital and St. Joseph’s Hospital lead to the formation of the Reykjavík Hospital (Sjúkrahús Reykjavíkur). Within the new organization’s administrative structure, Orthopedic Surgery established a department of its own and Casualty became an Emergency Department (ED) under the daily leadership of EM. Internal Medicine was also given an augmented role in the administration of the ED, bringing in more of their doctors in training but without added commitment on behalf of the specialist staff. In the same year, EM was included in a new version of the Icelandic government’s regulations defining which medical specialties were officially recognized in the country. All of this this created an opportunity for EM to exercise its newly won recognition but at the same time put significant burdens on the shoulders of a young specialty. EM bore the brunt of the daily operations and staffing of the ED which continued to be based on a combination of rotating residents and moonlighting senior staff from various specialties. It took until 1998 to hire enough dedicated staff to maintain two lines of specialist coverage during the day and one for the night shift. As soon as this was accomplished however and specialist physicians became directly involved in patient care, a notable improvement in the level of quality of services was achieved.

## National and international cooperation

The Icelandic Society for Emergency Medicine (ISEM) was founded 15 December 2000. There were about thirty members, many of whom were specialists in other fields of medicine or surgery who had reverted to a career in EM. Also among the founding members were doctors from other specialties who wanted to show their support for the concept and implementation of EM as a specialty. Jón Baldursson, MD, was elected as chairman and served in this position till 2002 when he was succeeded by Dr. Ólafur R. Ingimarsson. Today there are still around thirty members, but certified specialists in EM now make up the majority of the membership. The current chairman is Mikael S. Mikaelsson, MD.

As of 2016, ISEM is a member society of both the International Federation of Emergency Medicine (IFEM) and the European Society for Emergency Medicine (EuSEM). ISEM also participates in the informal network of the Nordic Federation for Emergency Medicine (NordFEM) which was initiated in 2012 in order to facilitate progress in the field of EM and cooperation between the Nordic societies for EM. Under the heading of NordFEM, meetings have been arranged during annual conferences of the participating societies for discussions on the development of EM.

An important challenge in building EM and getting medical students and recent graduates from medical school interested in EM is to have role models available. Having the first Nordic EM physician working in the ED was no doubt a key in building the specialty, but the progress was greatly aided by visiting EM physicians. During the early years, several formally trained EM physicians came to visit from abroad and spent days or weeks in the ED giving lectures, socializing with junior trainees and often working and providing an EM viewpoint on how to manage cases in the ED. The most notable contribution was that of Curtis P. Snook, MD, who is a specialist in both emergency medicine and medical toxicology. He did more than just visit Iceland, he moved from the U.S. to Iceland in 1995 with his family, learned to speak Icelandic and became the second certified specialist in EM in Iceland. He worked in Iceland for 7 years and was instrumental in modernizing both EM and toxicology services [12]. When he moved back to the U.S. in 2002 he was made Honorary Fellow of the ISEM.

A major step to advance this whole development was the international conference Emergency Medicine Between Continents, held in Reykjavik in June 2002 [13]. This meeting attracted about 300 participants, many of whom were leaders in emergency medicine on a global scale at that time. One of them referred to this event as “this Woodstock of emergency medicine”. The attention that the congress drew greatly helped EM gaining respect as an independent specialty within the Icelandic system. Mary Palmer, another EM and toxicology specialist from the US, was the leading force within the organizing committee for the conference and subsequently became the other first-time Honorary Fellow of ISEM.

## Emergency medicine training in Iceland

As mentioned earlier, the current Landspítali University Hospital was formed in year 2000. Again, the organizational turmoil generated by the merger created new opportunities for EM. The ED was given added responsibilities for patient care, bringing in a broader patient population which shaped a clinical forum for proper postgraduate training in EM. In year 2002, the ED started an official two-year post-internship training program in EM. This was based on a curriculum which was created by ISEM in January 2002 and subsequently acknowledged by the health care authorities. The program was aimed at providing core EM training intended as a foundation to build on for more advanced training abroad [14]. It was also stipulated in the curriculum that specialist physicians who were trained in other specialties could “grandfather” into the specialty of EM by working for 2 years in an ED with a teaching program in EM (in Iceland, only the University Hospital would fulfil this requirement) and for 6 months in anaesthesia/critical care, also in a department with a teaching program in these specialties. Some of our colleagues, especially those with a surgical background, had already fulfilled the latter requirement during their training in their earlier specialty. This approach was in part based on the Manifesto for EM in Europe, published by EuSEM in 1998 [15]. It has generally worked well and has helped to speed up the recruitment of physicians dedicated to EM in Iceland.

One way to incorporate the model of EM into a health care system is partnering with an external institution experienced in EM to teach and foster the development. This was done at Landspítali University Hospital from 2008 to 2010 in cooperation with Harvard University faculty of EM from the Beth Israel Deaconess Medical Center (BIDMC) group. The program included an onsite program director working clinically in the ED and overseeing lectures and a formal teaching program. In addition, visiting clinicians from BIDMC delivered concise courses on ultrasound, airway management and other critical issues for the development of EM. This helped greatly to improve the quality of training for resident physicians, but also reinforced the already defined pathway for “grandfathering”.

How postgraduate training of physicians is supervised varies significantly between different countries and systems. In the ED at Landspítali, close supervision of trainees has always been a key to improving the services provided, both in terms of clinical care delivered to the patients but also with regards to the teaching received by the trainees. The ED has 24/7 attending coverage and trainees are expected to present every case to the specialist physician. This is in stark contrast to the services provided by almost all other departments in the hospital where specialist physicians are rarely available in the ED and supervision outside of daytime hours largely is done over the phone. One of the typical problems encountered with establishing EM is that many of the decision makers from other departments in the hospital may have worked in the ED with minimal supervision years or decades ago, but are almost never seen in the department today. This may translate into those individuals thinking that they know how the ED functions and how it should be managed, whereas the reality is that they are often unfamiliar with the concepts and practice of modern EM.

To circumvent this problem, the ED at Landspítali has found regular case conferences to be very helpful in both improving care in the ED and strengthening collaboration with other departments in the hospital. These have not been in the traditional format of Morbidity and Mortality conferences commonly held in surgical departments, but have focused on cases that we can learn from, both things that have been handled exceptionally well and cases where the patients could have been managed better. These case conferences are conducted in cooperation with the ED nurses and are oriented around discussing the patients’ course in the ED according to available records. Every decision is discussed in the light of the information that was available at the time the decision was made. If an error occurred or the patient had a poor outcome, care is taken not to assign blame to staff members personally for the event. Rather, the discussion is focused on identifying every opportunity where an unfavorable outcome might have been prevented.

The cases discussed are grouped with regards to collaboration with other specialties in the hospital. Representatives from specialties involved each time as consulting and/or admitting services are invited to attend the meetings. The nature of EM is that we often have very limited information initially to make our diagnosis and start treatment. Over time, as more information is gathered, another picture of the patient’s problems may emerge. In general, this in turn may tempt physicians from other departments of the hospital to apply their “retrospectoscope” and unduly criticize the care provided in the ED [16]. The outcome of these case conferences has been that specialist physicians from other departments have often obtained a better understanding of how the patient’s course was in the in the ED, what was done and why. Also, not infrequently, they have realized that the cause of a problem was not the care provided by the ED staff, but rather incorrect, inappropriate or delayed treatment by their own department.

## Current situation

It was clear to the early leaders of EM in Iceland that gaining a critical mass of EM trained specialist physicians was a matter of priority. At the start of this decade, the ED at Landspítali finally reached the critical number of fully trained EM physicians to enable the final steps towards fully adopting the model of EM. Currently, about 70.000 patients per year visit the ED and during daytime there may be simultaneously up to four specialist emergency physicians on duty. Currently there are 25 specialist physicians in 18 full-time equivalents. Of those, 19 physicians have a specialist certification in EM, either after completing a full primary EM training or through a “grandfathering” program in EM.

The original curriculum of 2002 for EM training in Iceland was written in the ordinary fashion, mostly listing the things an EM physician needs to know [14]. This approach is however in our opinion not the most efficient one to advance the current standard of training. Both in the US and in the UK, post-graduate medical training has evolved more into documenting attending supervision of procedures, case-based discussions, history and physical taking, teaching, research and quality improvement projects and other elements of training to become an attending level physician. The goal of this approach is to ensure that the trainee demonstrates sufficient knowledge and skills to manage patients in real life settings. Adhering to such documentation of trainee progress also makes it easier to identify the trainee failing to progress in training. If the system contains a paper trail of inadequate knowledge or skills, the administrator is provided with the tool to effectively mentor the trainee on how to mend any deficiencies if possible. If the trainee fails to do so, this approach also provides the training system with means to block the trainee from becoming an incompetent specialist physician, which is an essential factor to ensure that patients receive safe and adequate care in any health care system.

To advance this element of training, the ED at Landspítali is currently adopting the specialist training program of the Royal College of Emergency Medicine (RCEM) in the UK. After reviewing the available curricula in use in other Nordic countries, the EuSEM curriculum and the training requirements of the American Board of Emergency Medicine, the curriculum by the RCEM was felt to be most suitable for use in Iceland.

The focus of the RCEM curriculum is not so much on listing what the trainee needs to know, but more on documenting that a specialist physician has actually observed the trainee interacting with patients, performing procedures and discussing cases in detail. Intricate to the RCEM system is the use of an online module to electronically document trainee progression; the ePortfolio.

A key function of the RCEM model of EM training is that it is trainee led. This means that it is the responsibility of the training program to provide teaching and supervision, but the trainee is responsible for documenting his or her progress through training by checking the required boxes. At the end of each year, the trainee must then pass an Annual Review of Competency Performance to be able to progress to the next year in training.

Starting in the fall of 2017, Landspítali is now adopting a combined three-year training program called the Acute Care Common Stem (ACCS). This is a dedicated pathway for early training in the specialties of emergency medicine, anesthesia, critical care and acute internal medicine. The trainees will rotate for 2 years through the clinical departments of all of these specialties obtaining basic level training in each area before moving on to a third year in the specialty they plan to eventually complete training in. In the UK, the ACCS has become the standard of basic training in EM and an option for those training in anesthesia, critical care and acute internal medicine. The aim of this program is to increase collaboration and mutual understanding of each other’s specialties for the physicians who generally manage the acutely ill patients in the hospital.

The plan is to offer almost full EM training in Iceland in the near future. Trainees will continue to be encouraged to do a part of their training abroad, as this is felt to be an important component of becoming a fully trained specialist.

In addition to full specialty training in EM, efforts have been made to bring in EM experience as a component of the training of general practitioners (GPs). This has been done through a dedicated 4 month rotation in the ED for every GP trainee with additional training time available for those interested. The ED at Landspítali has been accredited by the Australasian College of Emergency Medicine (ACEM) to provide up to 18 months of diploma-level training in EM, designed for GPs working in rural areas. A challenge for GPs with regards to obtaining adequate training and continuous education in the management of emergencies is that most available courses only provide coaching in a limited area, such as advanced cardiac or trauma life support, and the approach is often very hospital based. In an attempt to provide more appropriate training for rural GPs in the scarcely populated Iceland, a five-day course for rural GPs was organized and has been taught several times providing broad training in how to deal with emergencies in the pre-hospital setting, regardless of which organ specific specialty the emergency is traditionally considered a part of.

In Iceland, as in many other countries, hospital beds are becoming increasingly difficult to obtain for patients needing admission. With the number of elderly individuals in the society with chronic illness expected to rise significantly in the future years, there is a risk of overcrowding becoming a serious problem. EM in Iceland has already suffered the consequences of this. Over the last 2–3 years there has been a significant increase in the number of patients waiting in the ED at Landspítali at any given time because hospital beds are not immediately available. Currently the average waiting time from decision to admit to patient actually leaving the ED is 16 hours. This has caused a serious strain on the department and in turn has resulted in increased waiting times for patients arriving to the ED. If this problem will not be solved, there is a risk that overcrowding will eventually have a negative effect on training and job satisfaction of EM doctors and increase the risk of burnout. The ED can never have control over how many people need its services, the staff will always have to see all patients that seek care. It is therefore important for any hospital that adopts EM to make sure to also have efficient means of providing further care for patients who need admission. Since crowding has been shown to negatively affect the outcome of emergency patients, the ED can not and must not function as an overflow inpatient ward [17, 18].

## Academic activities in emergency medicine

Academic research provides the foundation of knowledge for most things done in medicine. Lessons learned from scientific evidence usually apply universally, meaning that it is possible for an ED to effectively provide up to date high quality emergency care without any research activity of its own. In the initial stages of adopting EM in Iceland, a focus was placed on providing direct clinical care and training junior doctors. As the specialty became more established, the emphasis on research activity was increased. A research unit in EM was jointly chartered by Landspítali and the University of Iceland in 2010.

At the Faculty of medicine of the University of Iceland, an Associate Professor in EM was appointed in 2003. This was Brynjólfur Mogensen MD who advanced to become the first Professor of EM in Iceland in 2016. All medical students have been exposed to EM by a mandatory 2 week rotation in the fourth year of the 6 year medical school. During the final year, the medical students have also received EM training in toxicology and completed a course on advanced life support provided in collaboration between the departments of EM, anesthesia and critical care.

## EM in Iceland and other Nordic countries

The geographical location of Iceland between Europe and North America and the tradition for generations to travel abroad for advanced medical training has helped adopting the EM model into the health care system more rapidly than has been feasible in the other Nordic countries. With further influences from English-speaking countries elsewhere in the world, Icelandic EPs have been given an even better opportunity to creatively invent their own cocktail of EM. It is suitable for the people of Iceland, but it is also acting as an interesting example of how a small country may successfully develop this new specialty. EM has however not been adopted in Iceland without a fight. In its initial years the specialty had to deal with a strong opposition from influential leaders of other departments. In the process of working through this some important lessons on how to adopt EM in the Nordic health care setting have been learned.

The European curriculum for EM was accepted in 2009 [19]. Thereafter, the development of the specialty has actively proceeded in our continent [20, 21]. We have already seen in Nordic countries how some organizational developments in the fields of EM have proven successful [22–27]. Icelandic EPs have shared their experiences with other colleagues in international meetings but also in congresses of national societies of EM and through the NordFEM. ISEM organized a two-day workshop in the fall of 2015, not to teach the medical details of EM but to focus on how to bring about the necessary system changes to make things happen in the Nordic environment. More information on the course content and all of the presentations can be found at www.emiceland.is. This information exchange has given new perspectives for EPs working in Finland and Sweden as they are building the new specialty but also for colleagues in Denmark and Norway, which recently have made decisions to establish an independent specialty of EM with a full-length training program.

## Conclusion

The evolution of EM in Iceland reflects the very same steps that have been taken in much larger countries. Hence, Iceland may be conceived as a historical laboratory of EM development, thus revealing successes as well as problems in a nutshell. These experiences may be valuable and helpful for physicians and nurses in other countries, when they are facing similar situations.

## Abbreviations

ACCS: Acute Care Common Stem
ACEM: Australasian College of Emergency Medicine
BIDCM: Beth Israel Deaconess Medical Center
ED: Emergency Department
EM: Emergency Medicine
EP: Emergency Physician
EuSEM: European Society for Emergency Medicine
GP: General Practitioner
IFEM: International Federation for Emergency Medicine
ISEM: Icelandic Society for Emergency Medicine
NordFEM: Nordic Federation for Emergency Medicine
RCEM: Royal College of Emergency Medicine
UK: United Kingdom
US: United States of America
